# Ferroelectric-assisted gold nanoparticles array for centimeter-scale highly reproducible SERS substrates

**DOI:** 10.1038/s41598-017-03301-y

**Published:** 2017-06-15

**Authors:** Xiaoyan Liu, Minoru Osada, Kenji Kitamura, Takahiro Nagata, Donghui Si

**Affiliations:** 1 College of Metallurgy and Materials Engineering, Chongqing University of Science and Technology, Chongqing Key Laboratory of Nano/Micro Composites and Devices, Chongqing, 401331 China; 2 International Center for Materials Nanoarchitectonics (WPI-MANA), National Institute for Materials Science (NIMS), Tsukuba, Ibaraki 305-0044 Japan; 3 Soft Matter and Interdisciplinary Research Center, College of Physics, Chongqing University, Chongqing, 400044 China

## Abstract

Assemble metal nanoparticles into various ordered structures with scale up to centimeter area is required to meet diverse needs of lab-on-a-chips and analytic components. Here, we present the uniform and high-yield fabrication of centimeter-scale gold nanoparticles (AuNPs) array for SERS substrates. Ferroelectric-assisted assembly of AuNPs line array is successfully fabricated by using a periodically poled LiNbO_3_ (PPLN) single crystal as a template. SNOM-Raman shows that the uniform assembly of AuNPs exhibits a high density of “hot spots” arising from strong electromagnetic (EM) field coupling induced by adjacent AuNPs. Quantitative analysis based on SERS detection describes an excellent reproducibility with an intensity variation less than 7% at 1649 cm^−1^ of Rhodamine 6G. SERS spectra combined with 3D-FDTD modelling indicate that the EM enhancement occurs at all three excitation wavelength of 515, 561 and 633 nm and the 561-nm-laser displays the strongest Raman enhancement with an enhancement factor in an order of 10^9^. The corresponding experimental and theoretical results present a new strategy to fabricate large-area, highly reproducible and sensitive SERS substrates for practical applications.

## Introduction

Surface-enhanced Raman spectroscopy (SERS) is a highly sensitive vibrational spectroscopy that allows for the detection of analytes at extremely low concentrations^1, 2^. The successful detection of a small amount of analyte by SERS is influenced by many factors. Arguably the most important of these factors is the SERS substrate. Applicable SERS substrates should possess mainly (1) large surface area to adsorb as many molecules to contribute to Raman signal, (2) abundant “hot spots” of metal nanostructures to enhance local electric fields and thus Raman signal^3, 4^, and (3) highly reproducible Raman signal over the large surface area. Many years of research have been devoted to creating and optimizing SERS substrates in order to meet these requirements^5–9^. Among them, well-ordered structures such as arrays, of nanoparticles have attracted a massive amount of interest due to their benefits over randomly aggregated nanoparticles that might hinder specific properties of the assembled nanoparticles, including plasmonic coupling effects^10^, optical bandgaps^11^, and metamaterial effects^12^. Electron beam lithography, nanosphere lithography, focused ion beam pattering, vacuum evaporation and soft-lithography are the most used techniques for assembly of metal arrays, however, they are limited by the high costs *i*.*e*. expensive equipment with complicated procedures, and the enormous difficulties to achieve an assembled structure over a large area^13–15^. In addition, the metal arrays assembled via these techniques are nearly planar and thereby have limited surface area. Recently, Q. Yang *et al*. prepared gold nanoparticles (AuNPs) spot array by a direct inkjet printing method^16^, J. Lee *et al*. fabricated silver nanoparticles (AgNPs) line array using anisotropic buckling templates^17^. The former can achieve a high reproducibility with a comparative sensitivity, but it has a limitation of fabrication of large surface area due to the limitation of the technique that is based on the controlling of printing AuNPs droplets and their drying process. The later, however, requires an additional process to transfer the assembled AgNPs array on the predefined templates to the desired flat substrate.

Ferroelectric materials possess spontaneous polarization that can be reversed by the application of an external electric field. Polarization inversion results in domain patterns with polar surfaces that exhibit surface-bound charges. LiNbO_3_ is a widely known ferroelectric material benefiting from its large spontaneous polarization (~75 µC/cm^2^) existing only along the crystallographic c-axis^18^. By applying an external electric field greater than the coercive field, domain structures exhibiting antiparallel polarization along the c-axis can be fabricated^19^. This results in domain patterns with positively-poled (+Z) and negatively-poled (−Z) domains that have different surface reactivities due to their different polarization orientation. This property potentially enables domain patterned LiNbO_3_ to act as templates for assembly of various functional nanostructures^20^. Studies have shown that, with a supra band gap illumination (>3.9 eV), photochemical reduction of Ag^+^ to Ag° from an aqueous solution occurs to +Z surfaces of domain patterns^21, 22^ and domain walls^23, 24^. Although the photodeposition of AgNPs on LiNbO_3_ has been reported in several publications, difficulties on fabrication of large-area well-ordered structures with nanoparticles in high density and good uniformity impede its applications in SERS. Recently, B. J. Rodriguez *et al*. developed a chemical patterning technique to fabricate periodically proton exchanged LiNbO_3_ (PPELN)^25^, and they demonstrated that the PPELN substrates possess different plasmonic properties with PPELN creating a stronger SERS signal relative to periodically poled LiNbO_3_ (PPLN) substrates^26, 27^. However, the PPELN substrates made by periodically proton exchanged method exhibit surface morphology with steps between the +Z and −Z domains at a height of 6–8 nm^27^, which could be a demerit as used for SERS substrates. Till now, to our knowledge, there is no report on fabrication of AuNPs array using ferroelectric LiNbO_3_ single crystals.

AuNPs and AgNPs are most often used as SERS substrates because of their great Raman enhancement properties. However, silver lacks chemical stability and thus, if oxidized, the nanoparticles have weak scattering and strong Ohmic losses. In contrast, AuNPs have attracted a lot of interests for practical SERS applications due to their higher stability. Here, we report an easy-to-control technique based on ferroelectric-assisted assembly of AuNPs line array as high-performance SERS substrates with excellent reproducibility at a centimeter-scale area. Further, this technique can be applied to assemble metal nanoparticles into various ordered structures, such as spot array with scale up to centimeter area (Fig. S1) to meet the diverse needs of lab-on-a-chips and analytic components. Consequently, this ferroelectric-assisted assembly presents a new strategy to fabricate large-area and high reproducible SERS substrates.

## Results and Discussion

**Figure 1:**
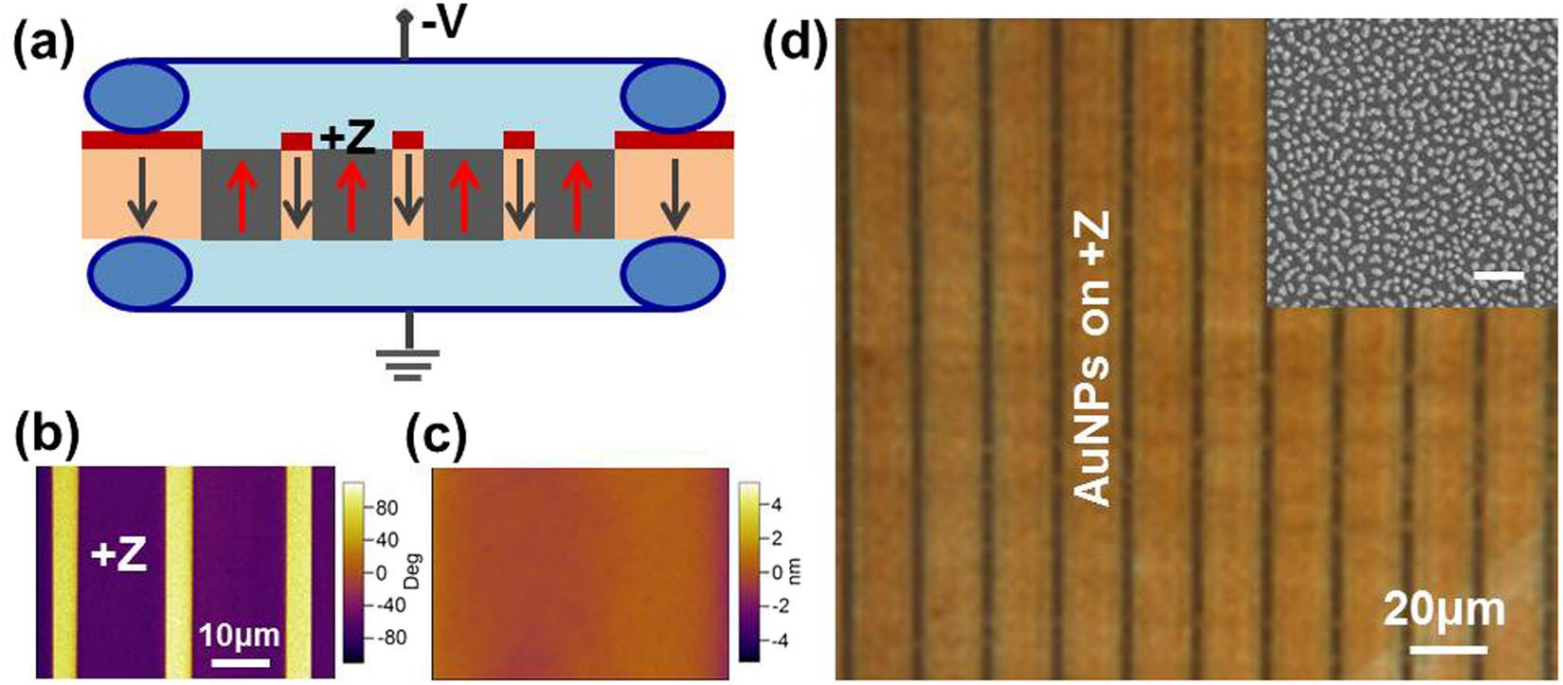
(**a**) Schematic of electric poling for fabrication of the PPLN. Photoresist (red blocks) with a periodical pattern was formed on the surface of negatively-poled LiNbO_3_ single crystal. An external negative voltage (>21 kV/mm, coercive field of the LiNbO_3_) was applied through a liquid electrode (light blue areas) to invert polarizations from negatively-poled ones (gray arrows) into positively-poled ones (red arrows). PFM phase (**b**) and topographic images (**c**) taken from the same area. (**d**) Dark field image of AuNPs array with AuNPs assembled onto +Z surfaces of the PPLN template. The insert is a representative SEM image showing morphology of AuNPs ca. 35 nm in average diameter. The scale bar in the insert is 200 nm.

PPLN is made by electric poling using congruent LiNbO_3_ single crystal wafer cut perpendicular to the crystallographic c-axis and completed at an optical level polishing^28^. The period of domain patterns can be manipulated at a range of a few micrometers to a few ten micrometers. Photochemically-induced assembly of AuNPs array on the PPLN was performed with optimized parameters of illumination and HAuCl_4_ aqueous solution. Figure 1 illustrates the procedure for assembling AuNPs array onto a flat PPLN template. This procedure involves mainly two steps: fabrication of flat PPLN template and assembly of AuNPs onto +Z domain surfaces of the template to form AuNPs array. In this work, in order to create AuNPs array with large areas, we fabricated PPLN with a width ratio of 4:1 of +Z and −Z domains at a period of 19.7 μm (Fig. 1a).

After poling, polarization property and surface flatness was confirmed by piezoresponse force microscope (PFM). Figure 1b is the phase image of the PPLN and the corresponding topographic image (Fig. 1c) obtained from the same area shows a highly smooth surface without height difference at domain boundaries. Photochemical deposition of AuNPs was performed immediately after the clean process by immersing the PPLN into an aqueous solution of HAuCl_4_ with a supra band gap illumination.

1 $${{\rm{H}}}^{+}+{{{\rm{AuCl}}}_{4}}^{-}+3{\rm{e}}\,\mathop{\longrightarrow }\limits^{\,nv}{\rm{Au}}+{{\rm{H}}}^{+}+4{{\rm{Cl}}}^{-}$$

The reaction associated with the salt solution and light irradiation is seen in Equation (1). Photo-excited electrons in LiNbO_3_ crystal move toward +Z domain surface due to the photogalvanic effect and are available for local reaction with HAuCl_4_^21, 29, 30^. The reduction of [AuCl_4_]^−^ to Au occurs preferentially over the +Z domains also because at which photo-excited electrons face no energy barrier from the conduction band of LiNbO_3_^30^. We used a white light delivered by a bundled fiber to realize carrier excitation. In order to assemble AuNPs array with nanoparticles at a high density and a certain dimension, a large number of preliminary experiments were performed through adjusting of concentration of HAuCl_4_ aqueous solution and illumination intensity. The illumination intensity varied by tuning the light output or the distance between the PPLN template and the output surface of the bundled fiber. A representative AuNPs array was displayed at a dark field image (Fig. 1d), the insert is a scanning electron microscope (SEM) image showing the AuNPs with high density and good uniformity. The average diameter (~35 nm) of AuNPs was calculated by measuring 30 particles of five different SEM images in each. Furthermore, surface morphology of the AuNPs was also demonstrated by the zoom-in ac-AFM image as shown in Fig. S2.

**Figure 2:**
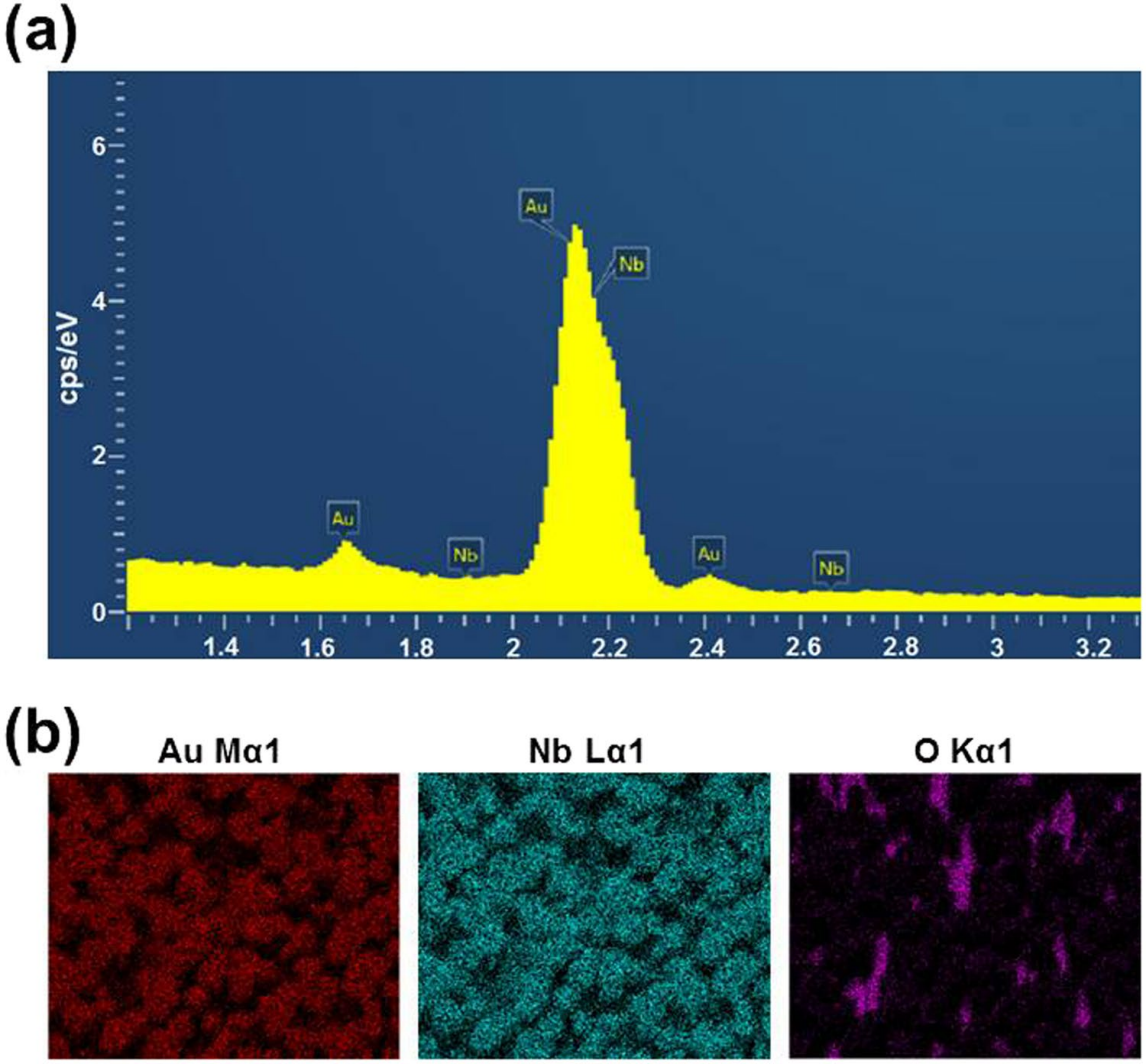
(**a**) EDS spectrum of AuNPs on the LiNbO_3_, containing detectable Au and Nb. (**b**) Element mapping of Au, Nb and O.

Energy-dispersive X-ray spectroscopy (EDS) was used to demonstrate the AuNPs assembled on +Z surfaces of the PPLN template. Figure 2(a) shows EDS spectrum of the AuNPs on the LiNbO_3_, containing detectable elements of Au and Nb. The peak at the highest energy corresponds to x-rays generated by emission from different energy-level shells (L, M) in Au and Nb due to their close overlaps of Au-Mα and Nb-Lα. Figure 2(b) shows element mapping of Au, Nb and O. Intentionally, the EDS was conducted in a selected region with low intensity of AuNPs in order to distinguish three different elements of Au, Nb and O. The mapping of O-Kα combining with that of Au-Mα and Nb-Lα demonstrates that the AuNPs were fabricated upon LiNbO_3_.

**Figure 3:**
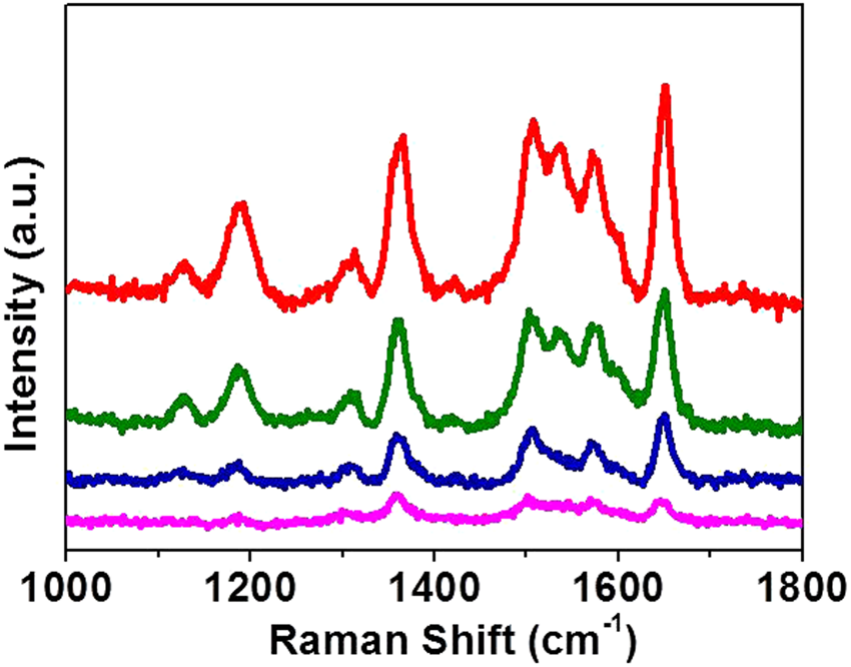
SERS spectra of 10^−5^–10^−8^ M R6G on the AuNPs array. The incident laser of 633 nm was used with the laser power of 1 µW and the laser diameter of 1 µm focused on the samples. The acquisition time was 10 s.

To evaluate the SERS performance of the assembled AuNPs array, Raman experiments were conducted employing Rhodamine 6G (R6G) as the probing molecule owing to its well-established vibrational features. Figure 3 shows the SERS spectra of R6G measured at a 633 nm laser with various concentrations ranging from 10^−5^–10^−8^ M on the AuNPs array. Most of the Raman bands match well with the characteristics of the Raman spectrum of R6G^31^. The bands at 1127 and 1183 cm^−1^ are assigned to the C-H in-plane bending mode, the band at 1310 cm^−1^ is assigned to the C−O−C stretching mode and the bands at 1362, 1509, 1580, and 1649 cm^−1^ are assigned to the C-C stretching modes. The signals are found to be monotonically decreasing with the decreased concentration, despite the low concentration of 10^−8^ M, it exhibited clearly the main Raman peaks. It should be mentioned that the 633 nm laser is not a best match with the AuNPs structure, which was confirmed later by wavelength dependence of SERS intensity, thus our fabricated substrate is expected to be able to detect probe molecules at a lower concentration (<10^−8^ M) by using an optimized laser. In addition, it was confirmed that all Raman bands of LiNbO_3_ single crystal are beyond the measuring range of 1000–1800 cm^−1^ of the probing molecule R6G (Fig. S3). Raman spectra of different probing molecules of p-aminothiophenol (PATP), 2-aminothiophenol, and methylene blue (MB) are shown in Fig. S4, further demonstrating SERS performance of the assembled AuNPs array.

For quantification, the enhancement factor (EF) was calculated using the expression

2 $${\rm{EF}}=[{I}_{{\rm{SERS}}}]/[{I}_{{\rm{normal}}}]\times [{C}_{{\rm{normal}}}]/[{C}_{{\rm{SERS}}}]$$

where *C*_normal_ and *I*_normal_ correspond to the concentration and peak intensity for the regular Raman measurement with 0.5 M R6G solution on a LiNbO_3_ wafer (Fig. S5), respectively; and *C*_SERS_ and *I*_SERS_ are the concentration and peak intensity for the SERS measurement with 10^−6^ M R6G molecules adsorbed on the AuNPs array, respectively. This calculation is based on the fact that the intensity of SERS is proportional to the number (or concentration) of molecules if the number of molecules is below a single-molecule layer^15^. The calculation was made based on the intensity of the carbon stretching mode at 1649 cm^−1^, the EF was calculated to be 5.8 × 10^8^. The high SERS sensitivity was possibly mainly attributed to the large electromagnetic (EM) field coupling at the junctions of AuNPs, as will be discussed further in the section of SNOM measurement and theoretical modelling. It should be noted that the interparticle distance among the adjacent AuNPs was much smaller than the laser-spot size in the Raman measurement, which ensures the contribution of narrow interparticle gaps to the EM field coupling.

**Figure 4:**
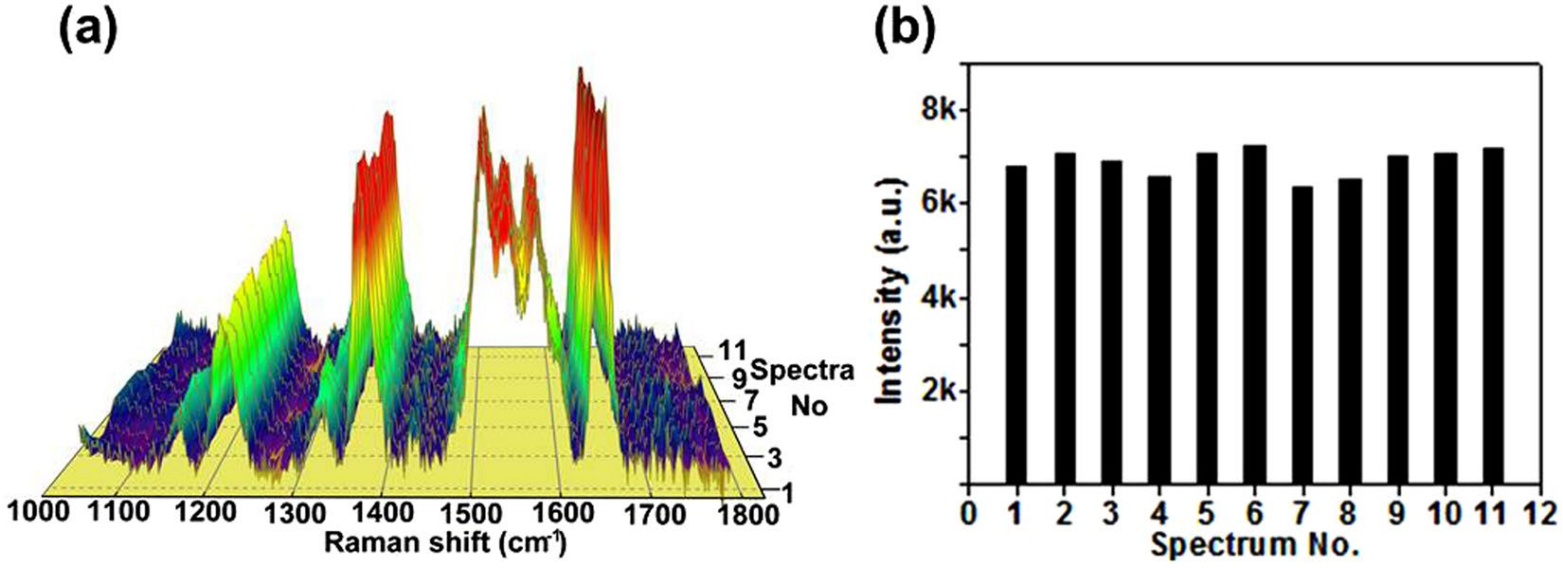
(**a**) Reproducibility of Raman spectra of 10^−6^ M R6G on the AuNPs array. Colors are assigned according to the relative intensity of the spectra. (**b**) Intensity distribution of the 1649 cm^−1^ peak in the 11 spectra.

Another important advantage of the AuNPs array is the homogeneous site enhancement distribution over centimeter-scale area, yielding improved reproducibility of Raman signals. Figure 4a shows the SERS spectra of 10^−6^ M R6G recorded at 11 randomly chosen spots across the AuNPs array. The spot-to-spot intensity variations of the characteristic 1649 cm^−1^ peak are quantitatively displayed in Fig. 3b, which shows that the total 11 data points exhibit an intensity variation less than 7%. The remarkable reproducibility suggests the presence of the uniformity of site enhancement distribution over the AuNPs array.

**Figure 5:**
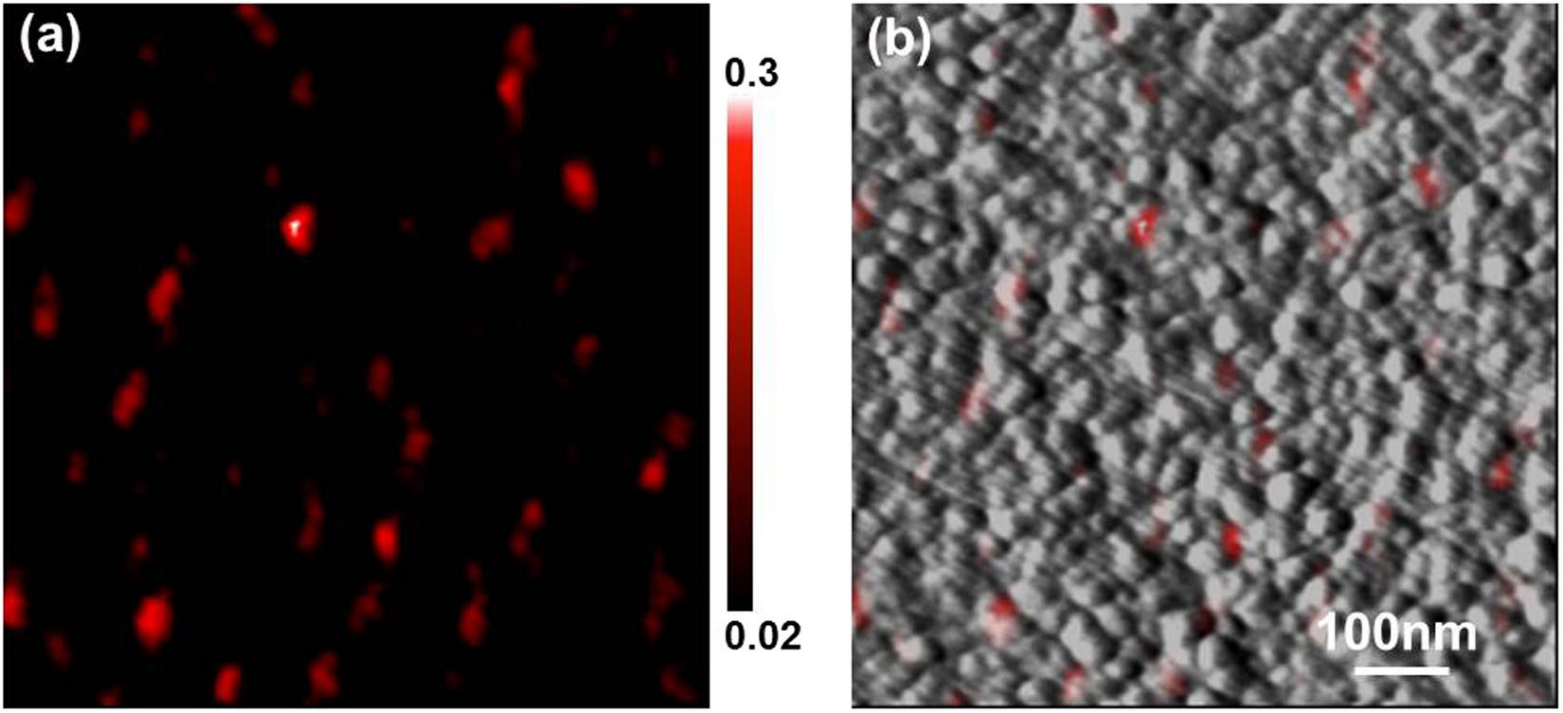
(**a**) SNOM-Raman mapping. (**b**) SNOM-Raman mapping overlaid on the corresponding topographic image.

SNOM enables studying sample optical properties on the nanometer scale and the technique has been widely used in plasmonics. In this work, a SNOM-Raman^32, 33^ was used to correlate the EM field with the surface topography of the AuNPs. Figure 5a and b show SNOM-Raman mapping and that overlaid on the corresponding topographic image of the AuNPs, respectively. Figure 5a demonstrates the uniform distribution of the EM field *i*.*e*. “hot spots”^34, 35^ over the AuNPs, while Fig. 5b visualizes the highly localized EM field at the interparticle gaps among the adjacent AuNPs, which contributes to the high SERS sensitivity of the substrate.

**Figure 6:**
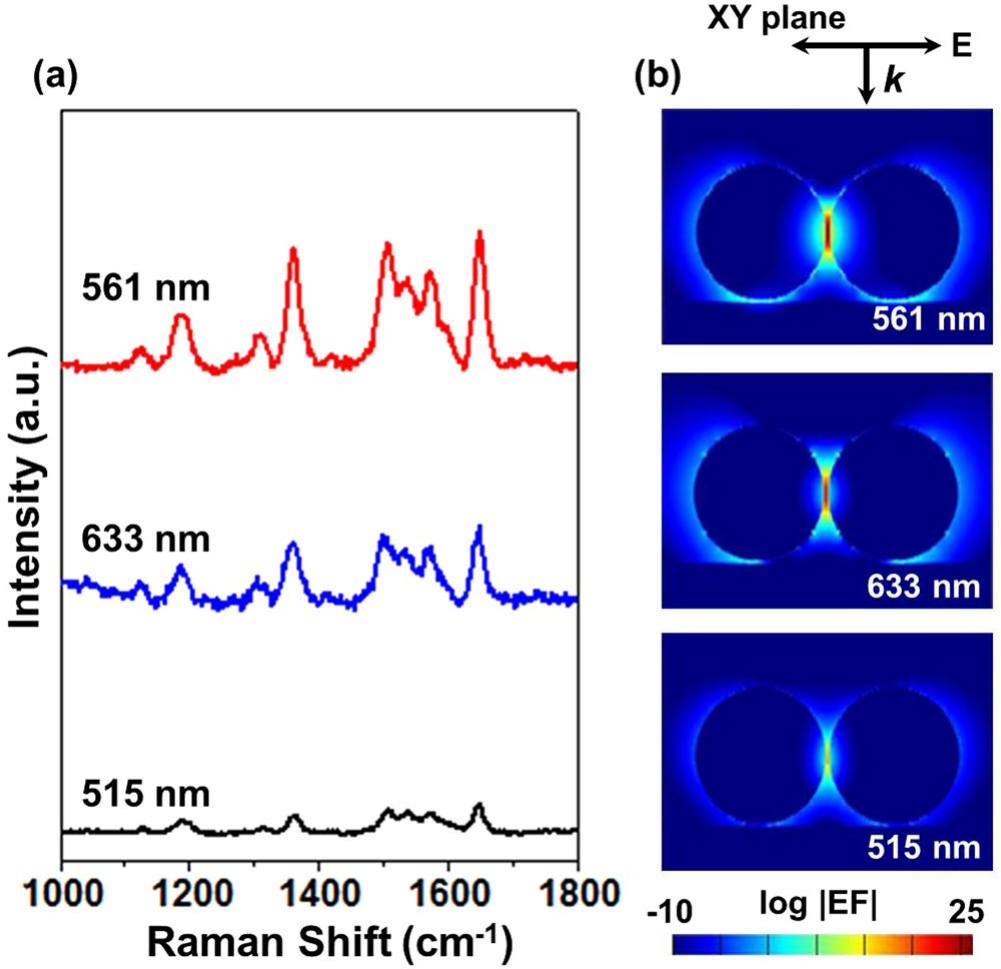
(**a**) SERS spectra of R6G at the excitation wavelengths of 515, 561 and 633 nm at the same power. (**b**) The FDTD calculated local electric field enhancement (log |EF|) of dimer AuNPs at the XY plane.

Because the SERS effect mainly arises from the plasmon resonance of the nanostructures, it is typically wavelength dependent. In this work, we examined the SERS properties of R6G on the AuNPs array with laser excitation at 515, 561, 633 nm. Theoretical modelling with 3D-FDTD was applied to calculate the electric field distribution of the AuNPs in terms of the laser wavelength. We built the theoretical model as the AuNPs displayed in the insert SEM image in Fig. 1d, where shows dimer configurations dominating the structure. The measured average diameter of 35 nm was used for modelling and the separation between two AuNPs was set at 1 nm roughly correspond to the size of R6G molecule. Figure 6a indicates that the EM enhancement was found at all three incident lasers and SERS spectra obtained at 561 nm provided the strongest Raman enhancement with an EF in the order of 10^9^. The theoretical modelling (Fig. 6b) shows accordingly that the magnitude of electric field for the plasmon resonance between AuNPs was obviously different with the assist of the incident laser, and the maximum enhancement was achieved at 561 nm, which is well consistent with the experimental SERS spectra. The corresponding theoretical and experimental results further demonstrated that the SERS of R6G molecules on the AuNPs array is mainly dominated by the EM effect. These results also imply that a higher sensitivity can be realized by optimizing nanostructures and laser wavelength.

## Conclusions

In summary, ferroelectric-assisted assembly of large-area AuNPs array is used as SERS substrates for sensitive molecule detection. The AuNPs array possesses uniform site enhancement distribution and is capable of quantitative analysis of R6G molecules with excellent reproducibility of less than 7% intensity variation at the major vibration. Corresponding experimental and theoretical results show that the electromagnetic fields significantly enhanced on the surface of AuNPs array with an enhancement factor in an order of 10^9^ at the excitation wavelength of 561 nm. Based on spontaneous polarization properties of ferroelectrics, this technique has advantages for assembling AuNPs into various ordered structures at a centimeter-scale large area. The totality of the results suggests ferroelectric-assisted AuNPs array to be a promising candidate of SERS substrates for practical SERS applications.

## Materials and Methods

### Preparation of PPLN template

The LiNbO_3_ samples were cut from a 0.3-mm-thick *Z*-cut LiNbO_3_ wafer (Oxide Ltd., Japan). Polarization inversion was performed using an electric poling technique with a patterned photoresist covered by a metal film on the −Z surface and a continuous liquid electrode of LiCl aqueous solution on the +Z surface. Periodically poled LiNbO_3_ (PPLN) was then diced into pieces in a size of 8 × 10 mm^2^ (Swing Ltd., Japan), and they were used as photodeposition templates after the clean process.

### PFM characterization of PPLN

The polarization property and surface flatness of the PPLN templates were measured using piezoresponse force microscope (PFM, Asylum Research MFP-3D). During the PFM imaging, a 2 V AC voltage was applied to a conductive cantilever (spring constant of 3.5 N/m and tip radius of 25 nm) while scanning the tip on the template surface. The PFM phase image provides information of polarization direction. The topography was recorded simultaneously with the PFM phase image via the MFP-3D controller.

### Fabrication of centimeter-scale AuNPs array

The PPLN templates were cleaned prior to the photodeposition via sonication for 20 min each in acetone, ethanol and Milli-Q water sequentially. The templates were placed into a small vessel made with an O-ring sealed upon a slide glass and that was filled with 300 μL of HAuCl_4_ aqueous solution. A spot light source (San-ei Electric) equipped with a 200 W xenon lamp was used for the illumination at a distance of 1.5 cm above the template surface for 5 min. The light was delivered by a bundled fiber with a spot diameter of 5 mm and a total intensity of 1.235 W/cm^2^ in the wavelength range of 200–400 nm. After the deposition, the substrates were immersed into Milli-Q water for 1 min and then blown dry with nitrogen.

### SEM and AFM measurements

Surface morphologies of AuNPs were obtained by using a scanning electron microscopy (SEM, JSM-7800E) and an atomic force microscopy (AFM, Asylum Research MFP-3D). The AFM was performed at ac-mode. The SEM was performed at an accelerating voltage of 5 kV and a working distance of 9.6 mm attached an energy-dispersive X-ray spectroscopy (EDS). In order to obtain information about containing elements, the samples don’t have any surface coating layer though it is generally used to enhance the electron conductivity during the scanning process.

### SERS measurements

R6G was used as a probe molecule for SERS measurements. The substrates were immersed in a 10^−5^–10^−8^ M R6G aqueous solution for 30 min to reach an adsorption/desorption equilibrium. Then, they were rinsed with Milli-Q water to remove any unabsorbed molecules, and dried with a stream of air. Raman spectroscopy was conducted using a Horiba-Jobin-Yvon Raman System T64000. A 100x (NA = 0.75) objective was used to focus the laser on a target area and to collect the backward scattering light from the sample surface. A spot size of 1 µm in diameter and excitation wavelength of 515, 561 and 633 nm from a diode laser at a power of 1 µW was used to irradiate the sample surfaces. The irradiation time for SERS spectra was 10 s.

### SNOM measurements

Scanning near-field optical microscope (SNOM) system, based on a commercial SNOM (Omicron, Twin-SNOM), is specially upgraded for spectroscopy purpose. The gold-coated fiber probe, forming the SNOM aperture, can be moved with nanometer accuracy to and from the surface. Single-mode fiber probes capable of linear polarized light output (~20%) were selected for use in this experiment. The SNOM probe is held within 10 nm of the sample surface using the shear-force feedback technique. An Ar^+^ laser (λ = 515 nm, ~1 µW) was used for an excitation source. Raman-scattered light was detected in a backscattering configuration using the reflective objective lens and then passed through a holographic notch filter (Kaiser, Super Notch-Plus) to remove elastically scattered light before being focused into the spectrometer. A thermo-cooled intensified CCD camera (Andor Technology, DV438) was used in conjunction with a Czerney-Turner spectrometer for the Raman signal detection.

### 3D-FDTD simulation

A three dimensional finite difference time domain (3D-FDTD) simulation (Lumerical Solutions, Inc.) was applied to calculate the electric field distribution of the AuNPs. The AuNPs were illuminated by normal incident light with its polarization along the X-direction (Fig. 5b). The separation between two AuNPs was set at 1 nm roughly correspond to the size of R6G molecule.

## Electronic supplementary material

Ferroelectric-assisted gold nanoparticles array for centimeter-scale highly reproducible SERS Substrates
