# Supplementary material for: Ferroelectric-assisted gold nanoparticles array for centimeter-scale highly reproducible SERS substrates
Source: Sci Rep. 2017 Jun 15;7:3630. doi: 10.1038/s41598-017-03301-y (PMC5472578; doi:10.1038/s41598-017-03301-y)
Supplement: Supplementary file 1 — Ferroelectric-assisted gold nanoparticles array for centimeter-scale highly reproducible SERS Substrates [file 41598_2017_3301_MOESM1_ESM.pdf]

Ferroelectric-assisted gold nanoparticles array for centimeter-scale highly reproducible SERS Substrates

Xiaoyan Liu*[1], Minoru Osada*[2], Kenji Kitamura [2], Takahiro Nagata[2] & Donghui Si[1,3]

[1]College of Metallurgy and Materials Engineering, Chongqing University of Science and Technology, Chongqing Key Laboratory of Nano/Micro Composites and Devices, Chongqing 401331, China

[2]International Center for Materials Nanoarchitectonics (WPI-MANA), National Institute for Materials Science (NIMS), Tsukuba, Ibaraki 305-0044, Japan

[3]Soft Matter and Interdisciplinary Research Center, College of Physics, Chongqing University, Chongqing, 400044, China

Correspondence and requests for materials should be addressed to X.Y.L. (email: xyliu@cqust.edu.cn) or M.O. (email: Osada.minoru@nims.go.jp)

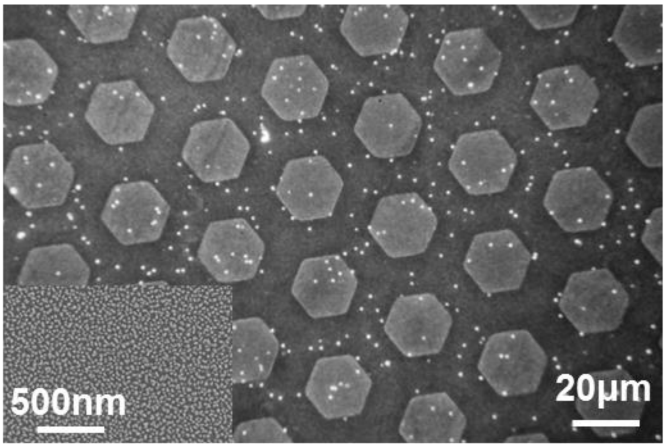

Figure S1 Dark field image of AuNPs dot array with AuNPs assembled onto +Z surfaces of $LiNbO_3$. The $LiNbO_3$ single crystal was previously subjected to a periodically polarization inversion at a domain pattern structured with positively-poled dot array. The insert SEM image shows morphology of AuNPs assembled upon dot domains.

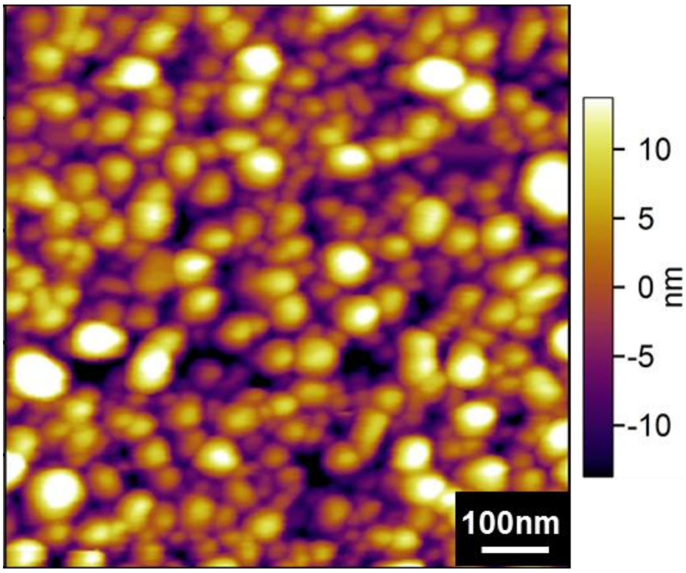

Figure S2 zoom-in ac-AFM image of AuNPs assembled over the +Z domains.

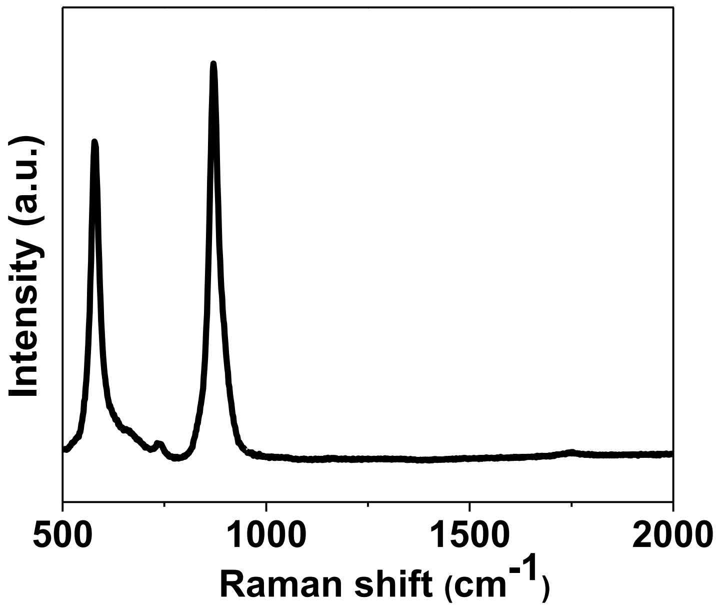

Figure S3 Raman spectra of $LiNbO_3$ single crystal.

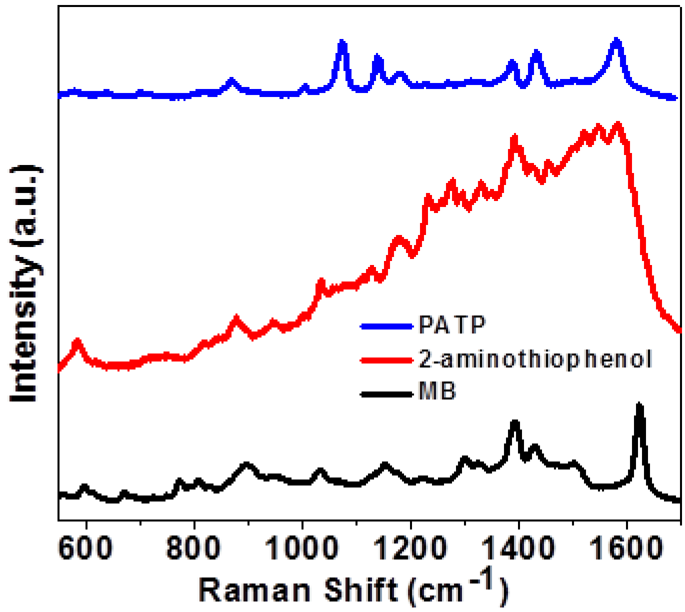

Figure S4 SERS spectra of 10^{-6} M *p*-aminothiophenol (PATP, blue), 2-aminothiophenol (red), and methylene blue (MB, black) on the AuNP arrays. The exciting laser of 633 nm was used with the laser power of 1 μW and the laser diameter of 1 μm focused on the samples. The acquisition time was 10 s.

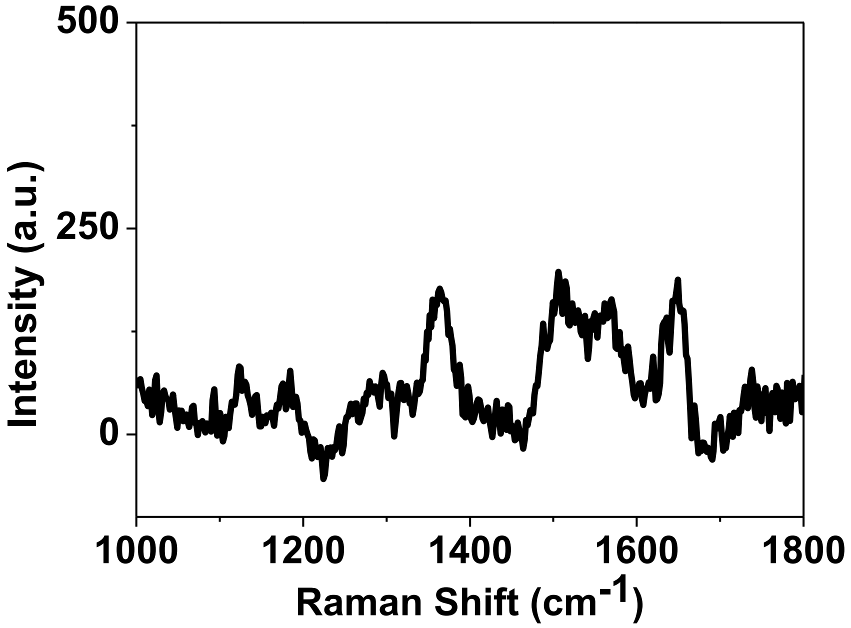

Figure S5 Raman spectra of 0.5 M R6G solution. The incident laser of 633 nm was used with the laser power of 1 μW and the laser diameter of 1 μm focused on the sample.
